# Supplementary material for: Host-directed therapy with amiodarone in preclinical models restricts mycobacterial infection and enhances autophagy
Source: Microbiol Spectr. 2024 Jun 25;12(8):e00167-24. doi: 10.1128/spectrum.00167-24 (PMC11302041; doi:10.1128/spectrum.00167-24)
Supplement: Supplemental material — Fig. S1 to S5; Table S1. [file spectrum.00167-24-s0001.docx]

# Supplementary material

## **Table S1: Zebrafish lines**


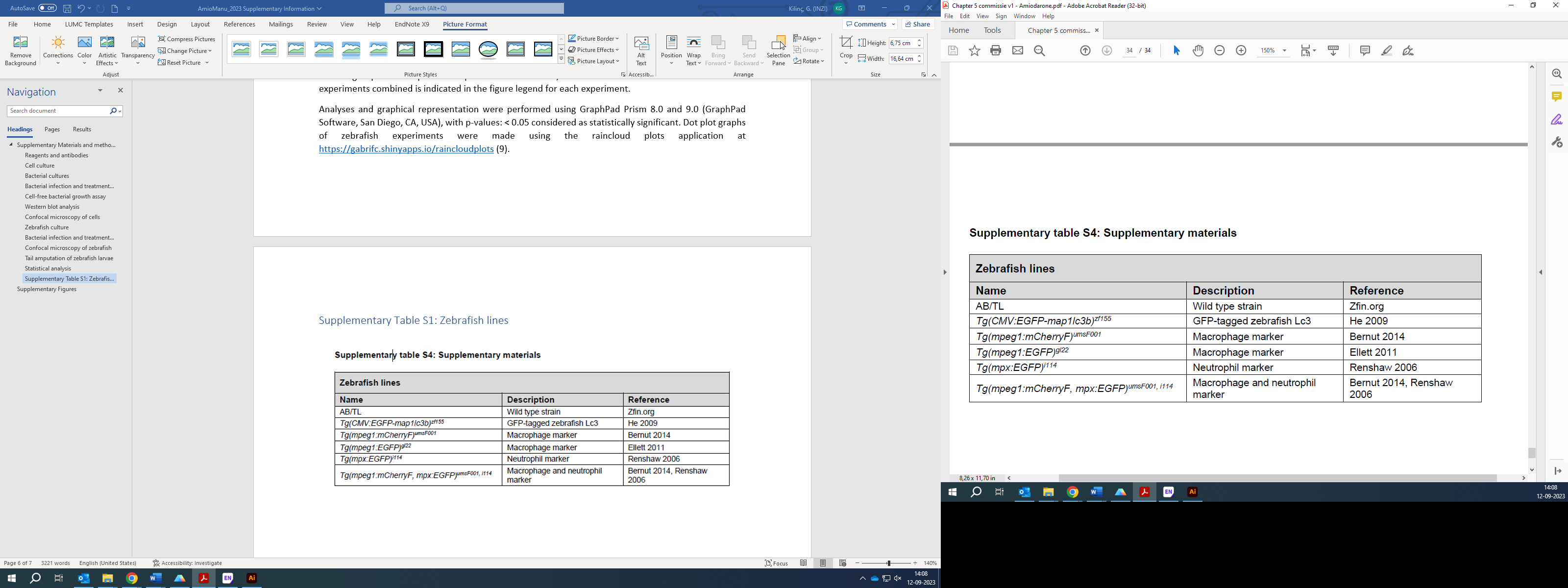

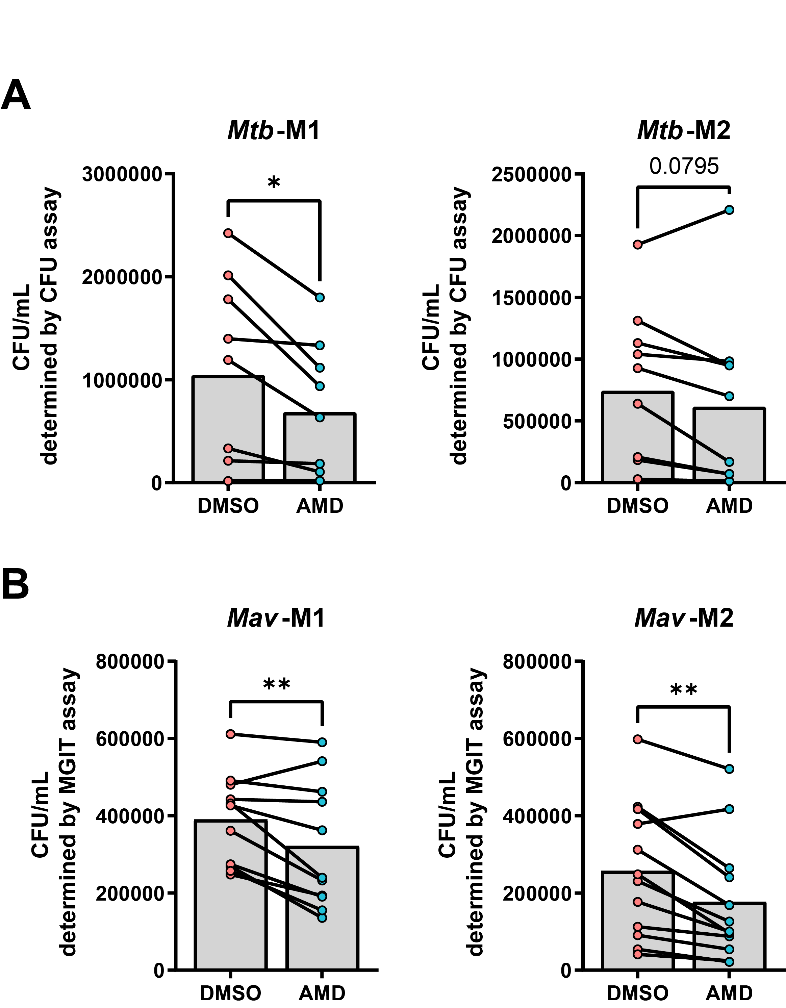


## **Figure S1. Identification of amiodarone as host-directed therapeutic for mycobacterial infections in primary human macrophages.**

**(A)** *Mtb* H37Rv-infected M1 and M2 macrophages were treated 24 hours with 10 µM amiodarone or an equal volume of vehicle control DMSO. Cells were subsequently lysed and bacterial survival was determined by CFU assay. Data represent the mean from different donors (n=9 or 10). Dots represent the mean from triplicate wells of a single donor. Statistical significance was tested using a paired t-test.

**(B)** *Mav*-infected M1 and M2 macrophages were treated 24 hours with 10 µM amiodarone or an equal volume of vehicle control DMSO. Cells were subsequently lysed and bacterial survival was determined by MGIT assay. Data represent the mean from different donors (n=11 or 12). Dots represent the mean from triplicate wells of a single donor. Statistical significance was tested using a paired t-test.

* = p<0.05 and ** = p<0.005.


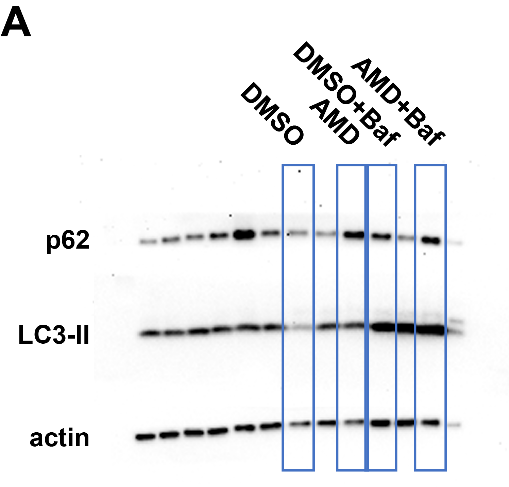


## **Figure S2. Protein levels of autophagy markers in primary human macrophages treated with amiodarone, in the absence or presence of bafilomycin.**

**(A)** Western blot analysis of autophagy markers in M2 macrophages treated for 24 hours with 10 µM amiodarone or an equal volume of vehicle control DMSO (0.1% v/v) in the presence or absence of bafilomycin A1 (Baf) (10 nM) during *Mav* infection. Shown are blots for p62, LC3-II and actin from one representative donor out of six donors tested. The boxed lanes represent the lanes shown in Fig. 2A, whereas unboxed lanes contain samples that are not relevant for this study.


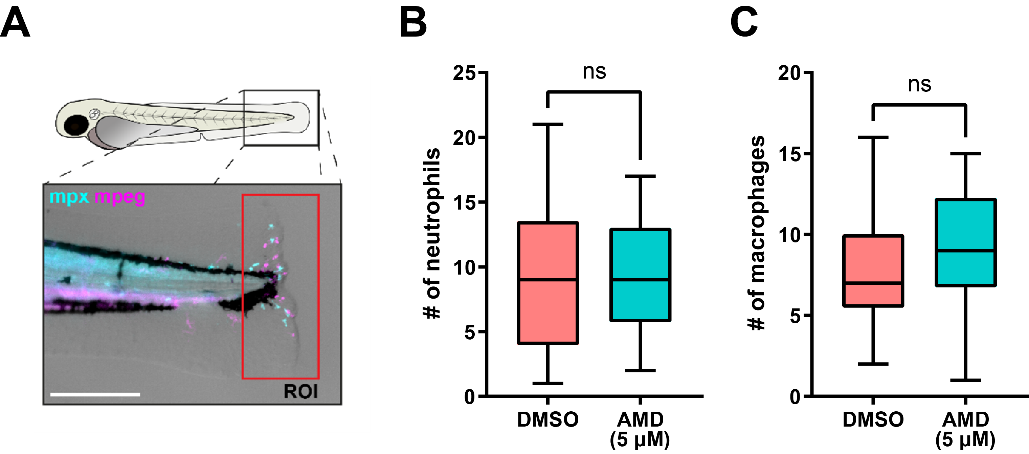


## **Figure S3. Amiodarone did not affect numbers of neutrophils and macrophages at the site of inflammation.**

**(A)** Leukocyte migration assay of mpeg1:mcherryF/mpx:GFP double transgenic zebrafish larvae treated with 5 μM of Amiodarone or control (DMSO at equal v/v). Treatment was started at 1 dpf and larvae were anesthetized and leukocyte migration was induced by tail amputation at 3 dpf. Representative stereo fluorescence images of leukocyte migration towards the injury (4 hours post-amputation) are shown. Cyan shows neutrophils (mpx:GFP) and magenta shows macrophages (mpeg1:mCherryF). The region of interest (ROI) indicates the area for quantification of leukocyte migration. Scale bar annotates 220 μM.

**(B-C)** Quantification of A, showing the number of migrated neutrophils (B) or macrophages (C). Boxplots with 95% confidence intervals are shown and the black line in the boxplots indicates the group median. Statistical analysis was performed using a Mann-Whitney test.

Ns: non-significant.


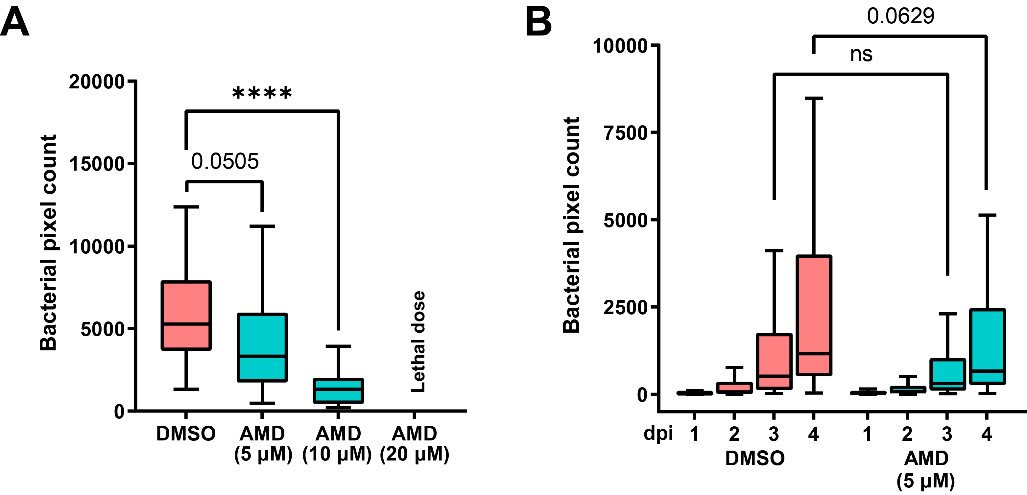


## **Figure S4. Amiodarone restricts *Mmar* infection in a host-directed manner.**

**(A)** mWasabi-expressing *Mmar*-infected zebrafish larvae were treated with increasing doses of amiodarone (5, 10 and 20 μM) or vehicle control DMSO. Treatment was started at 1 hpi and bacterial pixel counts were quantified at 4 dpi. Data of 2 independent experiments were combined (n= 39-42 per group). Boxplots with 95% confidence intervals are shown and the black line in the boxplots indicates the group median. Statistical significance was tested using a Kruskal-Wallis with Dunn’s multiple comparisons test.

**(B)** mWasabi-expressing *Mmar*-infected zebrafish larvae were treated with 5 μM of amiodarone or vehicle control DMSO. Treatment was started at 1 hpi and larvae were anesthetized at 1, 2, 3 and 4 dpi for quantification of by imaging. Data of 2 experimental repeats were combined (n= 65-70 per group). Boxplots with 95% confidence intervals are shown and the black line in the boxplots indicates the group median. Statistical significance was tested using a Kruskal-Wallis with Dunn’s multiple comparisons test.

Ns: non-significant and **** = p<0.0001.


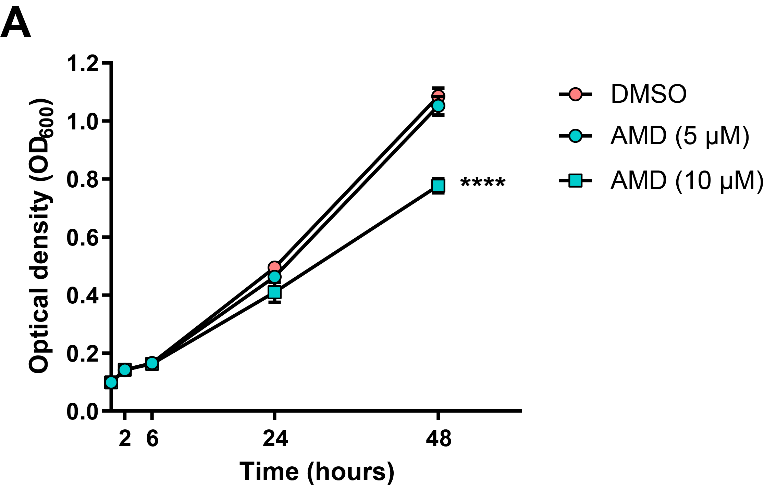


## **Figure S5. Growth of *Mmar* in liquid culture was not affected after exposure to 5 μM amiodarone.**

**(A)** *Mmar* growth in liquid culture during treatment with 5 or 10 μM of amiodarone or control (DMSO at equal v/v) up to assay endpoint, day 2. Lines depict mean ± standard deviation of 2 experiments. Statistical significance of treatment versus control treatment was tested using a two-way ANOVA with Dunnett’s multiple comparisons test.

**** = p<0.0001.
